# Synchronized dynamics of cortical neurons with time-delay feedback

**DOI:** 10.1186/1753-4631-1-2

**Published:** 2007-07-05

**Authors:** Alexandra S Landsman, Ira B Schwartz

**Affiliations:** 1 US Naval Research Laboratory, Code 6792, Nonlinear Systems Dynamics Section, Plasma Physics Division, Washington, DC 20375, USA

## Abstract

The dynamics of three mutually coupled cortical neurons with time delays in the coupling are explored numerically and analytically. The neurons are coupled in a line, with the middle neuron sending a somewhat stronger projection to the outer neurons than the feedback it receives, to model for instance the relay of a signal from primary to higher cortical areas. For a given coupling architecture, the delays introduce correlations in the time series at the time-scale of the delay. It was found that the middle neuron leads the outer ones by the delay time, while the outer neurons are synchronized with zero lag times. Synchronization is found to be highly dependent on the synaptic time constant, with faster synapses increasing both the degree of synchronization and the firing rate. Analysis shows that pre-synaptic input during the inter-spike interval stabilizes the synchronous state, even for arbitrarily weak coupling, and independent of the initial phase. The finding may be of significance to synchronization of large groups of cells in the cortex that are spatially distanced from each other.

## 1 Background

There is a significant amount of research showing that spike coincidence of neurons encodes information. Singer and colleagues showed not only that networks of neighboring neurons tend to become synchronized, but that tight synchronization occurs at the opposite hemispheres of the brain, in the presence of significant (6 to 8 milliseconds propagation delays) [1]. Zero time-lag synchronization of neural activity has been involved in such important phenomena as ability to recognize objects (by binding different attributes) [2,3], olfactory discrimination [4], and has even been proposed as one of the neural correlates of consciousness [5]. It was found that visuomotor integration is associated with synchronization of signals recorded from the visual and parietal, and motor and parietal areas of the awake cat [6,7].

While much of analysis focuses on network synchrony in the absence of time delays, delays are common in neural networks and there is evidence that under certain parameters [8] or coupling architectures, delays may actually contribute to synchronization. In a recent issue of Science [9], a short article describes a recent laser experiment [10] of mutually coupled lasers in a row. It is noted that when only two lasers are coupled, there is a lag in their phases equal to the amount of time it takes light to pass between them. However, when a third laser is added, the outputs of the outer lasers show zero time-lag synchronization. It has been suggested by Wolf Singer that this phenomena could shed light on synchronization of nerve signals in the brain in the presence of delays [9]. In particular, researchers indicate that this zero-lag synchronization effect in three coupled oscillators could clarify how the hemispheres of the brain synchronize [9].

With this in mind, we propose a three neuron coupling scheme, analogous to the three laser experiment described above. The synchronization phenomena of the three laser system has been recently analyzed [11], elucidating the conditions under which synchronization occurs. It was shown analytically and confirmed with numerics that increasing the delays improves synchronization of the outer lasers [11]. As will be shown in the present paper the dependence of synchronization on parameters is somewhat different in the three coupled neurons case, where it is actually the synaptic time constant, rather than delays in propagation that are important for synchronization of the outer neurons. It is perhaps not surprising that the mechanism behind synchronization in this three neuron model is different, since dynamics at each synapse are modeled by two differential equations, rather than much simpler linear coupling of the three laser scheme.

While the basic coupling architecture we choose to consider is the same as the above described laser experiment, in that we have a symmetric system of three mutually delay coupled oscillators, the projections from the middle to the outer neurons are somewhat stronger (with faster synaptic time constant) than the coupling from the outer to the middle neuron. This difference in coupling strength and synaptic time constant was inspired by the hierarchical structure of many networks in the brain, which to a first approximation is a feed-forward network, modulated by a weaker modulatory feedback [12]. For example, there is a strong forward projection from the thalamic LGN area to V1 or from V1 to MT, with weaker modulatory feedback projections that modulate the magnitude of the cell's response [3]. It has been suggested that exessively strong mutually coupled loops in the brain would promote uncontrollable oscillations, such as in epilepsy [12,13].

The present paper investigates a simple model for synchronization of cortical cells receiving a common time-delayed input from a different area of the brain, such as the case with projections of pyramidal cells to other cortical areas or the thalamus to the cortex, and sending a weaker time-delayed feedback. The central dynamical question addressed considers when the synchronous behavior of such a neural network is stable, particularly in regard to the synaptic coupling strengths, and the synaptic time constant. We find that shorter synaptic time constant of the target cells promotes synchronization, and even increases the firing rate, for the same strength of input.

**Figure 1:**
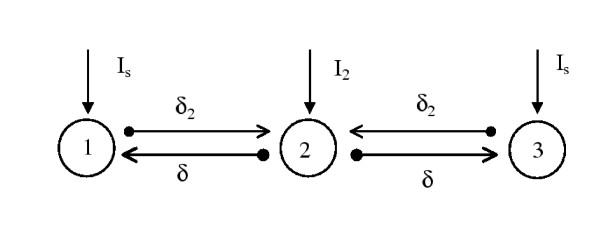
The basic model of three synaptically coupled neurons. The strength of the coupling from the middle to the outer neurons is given by *δ *and from the outer to the middle by *δ*_2_. The injected currents are shown. All synaptic coupling has propagation delay of *τ*_*d *_and a synaptic time constant of *τ*_*syn *_and *τ*_*syn*2 _for the outer and the middle neurons, respectively.

The paper is organized as follows: In Section 2, the basic model is set up, (see Fig. 1) and presented along with numerical results that show the dependence of synchronization and firing rate on the strength of coupling and synaptic conductance. Section 3, analyzes the synchronization observed for short synaptic times by linearizing about the dynamics of two nearby trajectories. It shows that fast synaptic input tends to synchronize two nearby trajectories, regardless of their phase with respect to the input signal. Section 4 concludes and summarizes.

## 2 Basic Model and Numerics

Neurons can be largely divided into two classes, dependent on their spiking properties. Class I neurons can be stimulated to fire at an arbitrarily low frequency, due to a saddle-node bifurcation, with increasing frequency as the magnitude of the stimulating current increases. Class II neurons, on the other hand, only begin to fire at relatively high frequency, with their limit cycle resulting from a subcritical Hopf bifurcation. Class II neurons are well represented by the squid axon, while a large majority of the mammalian neurons are of the Class I type. Dynamics of a human neo-cortical neuron in the absence of synaptic connections are well approximated by the following Class I neuron equations: [14]

**Figure 2:**
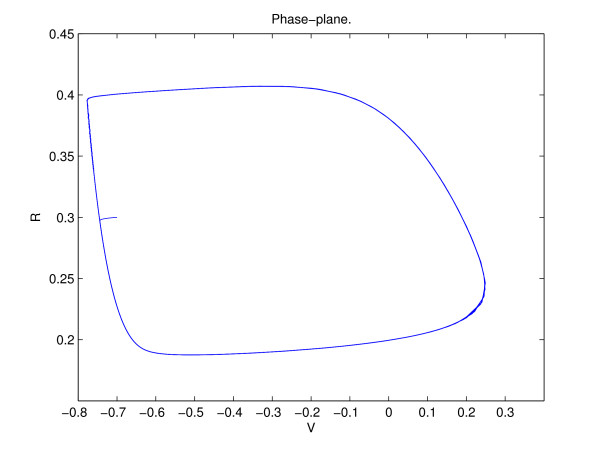
Limit cycle of an uncoupled cortical neuron, given by Eq. (1). *I *= 0.5 nA.

**Figure 3:**
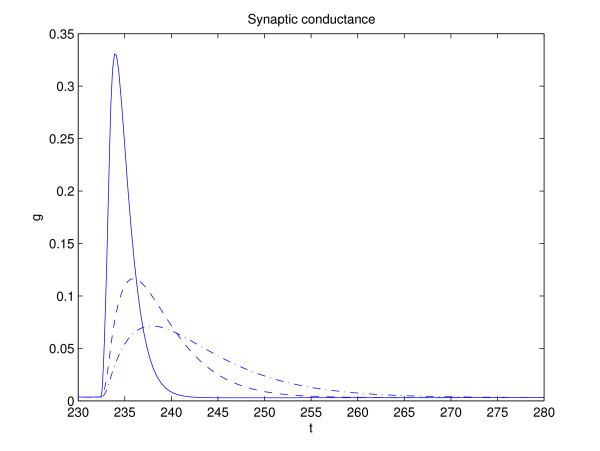
Conductance, *g*, after a single pre-synaptic spike. *τ*_*syn *_= 1, 3, and 5 ms.

dVdt=−{17.81+47.58V+33.8V2}(V−0.48)−26R(V+0.95)+I=F(V,R)+IdRdt=1τR(−R+1.29V+0.79+3.3(V+0.38)2)=G(V,R),
 MathType@MTEF@5@5@+=feaafiart1ev1aaatCvAUfKttLearuWrP9MDH5MBPbIqV92AaeXatLxBI9gBaebbnrfifHhDYfgasaacH8akY=wiFfYdH8Gipec8Eeeu0xXdbba9frFj0=OqFfea0dXdd9vqai=hGuQ8kuc9pgc9s8qqaq=dirpe0xb9q8qiLsFr0=vr0=vr0dc8meaabaqaciaacaGaaeqabaqabeGadaaakeaafaqabeGabaaabaWaaSaaaeaacqWGKbazcqWGwbGvaeaacqWGKbazcqWG0baDaaGaeyypa0JaeyOeI0Iaei4EaSNaeGymaeJaeG4naCJaeiOla4IaeGioaGJaeGymaeJaey4kaSIaeGinaqJaeG4naCJaeiOla4IaeGynauJaeGioaGJaemOvayLaey4kaSIaeG4mamJaeG4mamJaeiOla4IaeGioaGJaemOvay1aaWbaaSqabeaacqaIYaGmaaGccqGG9bqFcqGGOaakcqWGwbGvcqGHsislcqaIWaamcqGGUaGlcqaI0aancqaI4aaocqGGPaqkcqGHsislcqaIYaGmcqaI2aGncqWGsbGucqGGOaakcqWGwbGvcqGHRaWkcqaIWaamcqGGUaGlcqaI5aqocqaI1aqncqGGPaqkcqGHRaWkcqWGjbqscqGH9aqpcqWGgbGrcqGGOaakcqWGwbGvcqGGSaalcqWGsbGucqGGPaqkcqGHRaWkcqWGjbqsaeaadaWcaaqaaiabdsgaKjabdkfasbqaaiabdsgaKjabdsha0baacqGH9aqpdaWcaaqaaiabigdaXaqaaGGaciab=r8a0naaBaaaleaacqWGsbGuaeqaaaaakmaabmaabaGaeyOeI0IaemOuaiLaey4kaSIaeGymaeJaeiOla4IaeGOmaiJaeGyoaKJaemOvayLaey4kaSIaeGimaaJaeiOla4IaeG4naCJaeGyoaKJaey4kaSIaeG4mamJaeiOla4IaeG4mamJaeiikaGIaemOvayLaey4kaSIaeGimaaJaeiOla4IaeG4mamJaeGioaGJaeiykaKYaaWbaaSqabeaacqaIYaGmaaaakiaawIcacaGLPaaacqGH9aqpcqWGhbWrcqGGOaakcqWGwbGvcqGGSaalcqWGsbGucqGGPaqkcqGGSaalaaaaaa@94F5@

where *V *and *R *are voltage and recovery variables, respectively, and *τ*_*R *_= 5.6 ms. The *dR */*dt *equation is written as a sum of a linear term, for the normal *N a*^+ ^and *K*^+ ^currents, and a quadratic terms in *V *to approximate the transient potassium current contributions [15]. The above model has been optimized to provide an accurate quantitative fit to the shape of a regular spiking neuron potentials obtained from human neocortical neurons [16]. Figure 2 shows the limit cycle of a cortical neuron in Eq. (1) when the applied current, *I*, is above the bifurcation value, resulting in a saddle-node bifurcation. Due to a quadratic term in the recovery variable, the spike rate of a cortical neuron can be arbitrarily low, for low currents, and increases as *I *is increased. The above neurons can be coupled by adding an additional term to *dV */*dt *in Eq. (1) proportional to *g *(*V *- *E*_*syn*_), where *g *is the synaptic conductance variable, *E*_*syn *_= 0 for excitatory synapses and -0.92 for inhibitory. The synaptic conductance, *g*, is obtained from the following equations, commonly used for synaptic coupling [14],

dfdt=1τsyn(−f+Hstep(Vpre−Ω))dgdt=1τsyn(−g+f)
 MathType@MTEF@5@5@+=feaafiart1ev1aaatCvAUfKttLearuWrP9MDH5MBPbIqV92AaeXatLxBI9gBaebbnrfifHhDYfgasaacH8akY=wiFfYdH8Gipec8Eeeu0xXdbba9frFj0=OqFfea0dXdd9vqai=hGuQ8kuc9pgc9s8qqaq=dirpe0xb9q8qiLsFr0=vr0=vr0dc8meaabaqaciaacaGaaeqabaqabeGadaaakeaafaqabeGabaaabaWaaSaaaeaacqWGKbazcqWGMbGzaeaacqWGKbazcqWG0baDaaGaeyypa0ZaaSaaaeaacqaIXaqmaeaaiiGacqWFepaDdaWgaaWcbaGaem4CamNaemyEaKNaemOBa4gabeaaaaGccqGGOaakcqGHsislcqWGMbGzcqGHRaWkcqWGibasdaWgaaWcbaGaem4CamNaemiDaqNaemyzauMaemiCaahabeaakiabcIcaOiabdAfawnaaBaaaleaacqWGWbaCcqWGYbGCcqWGLbqzaeqaaOGaeyOeI0IaeuyQdCLaeiykaKIaeiykaKcabaWaaSaaaeaacqWGKbazcqWGNbWzaeaacqWGKbazcqWG0baDaaGaeyypa0ZaaSaaaeaacqaIXaqmaeaacqWFepaDdaWgaaWcbaGaem4CamNaemyEaKNaemOBa4gabeaaaaGccqGGOaakcqGHsislcqWGNbWzcqGHRaWkcqWGMbGzcqGGPaqkaaaaaa@63FC@

where *H*_*step *_(*x*) = 1, if *x *> 0 and zero if *x *< 0, *V*_*pre *_is the voltage of the presynaptic neuron and *τ*_*syn *_is the synaptic conductance time constant. In numerical simulation, Ω = -0.20 mV was chosen [14]. The reason for using two synaptic equations is that, depending on *τ*_*syn*_, the conductance will peak after *V*_*pre*_, continuing to depolarize the membrane after the end of the presynaptic spike (see Figure 3). This type of response is consistent with physiological data. For brief stimulus spike at *t *= 0, the two synaptic equations produce a response proportional to [13]

g=(t/τsyn2)exp(−t/τsyn)
 MathType@MTEF@5@5@+=feaafiart1ev1aaatCvAUfKttLearuWrP9MDH5MBPbIqV92AaeXatLxBI9gBaebbnrfifHhDYfgasaacH8akY=wiFfYdH8Gipec8Eeeu0xXdbba9frFj0=OqFfea0dXdd9vqai=hGuQ8kuc9pgc9s8qqaq=dirpe0xb9q8qiLsFr0=vr0=vr0dc8meaabaqaciaacaGaaeqabaqabeGadaaakeaacqWGNbWzcqGH9aqpdaqadaqaaiabdsha0jabc+caVGGaciab=r8a0naaDaaaleaacqWGZbWCcqWG5bqEcqWGUbGBaeaacqaIYaGmaaaakiaawIcacaGLPaaaieGacqGFLbqzcqGF4baEcqGFWbaCdaqadaqaaiabgkHiTiabdsha0jabc+caViab=r8a0naaBaaaleaacqWGZbWCcqWG5bqEcqWGUbGBaeqaaaGccaGLOaGaayzkaaaaaa@4970@

Figure 3 shows *g *for different values of *τ*_*syn*_. In each case, the area under the curve is the same (equal to one), with *τ*_*syn *_determining the width of the post-synaptic spike.

Using Eqs. (1) and (2) we can now set up the basic model of three mutually coupled neurons. The basic set-up is shown in Figure 1, where 3 neurons are coupled in a line. The middle neuron sends an excitatory time-delayed signal to the two outer neurons which are coupled to the middle one via weaker delayed coupling with a longer synaptic time constant. This model reflect the typically observed hierarchy in the brain (described in the introduction), where the neurons in one brain area, such as the thalamus, send strong excitatory connections to cortical neurons, receiving weaker modulatory feedback. Since the equations of the outer neurons are identical (resulting from a fit with experimental data) and they receive the same synaptic input from the middle cell, their synchronization would indicate that a group of cortical neurons with the same type of synaptic coupling will fire synchronously when subjected to a particular synaptic input. Using Eq. (1), the equations for the coupled neurons are given by:

dVidt=F(Vi,Ri)+Ii−δigi(Vi−Esyn)dRidt=g(Vi,Ri)
 MathType@MTEF@5@5@+=feaafiart1ev1aaatCvAUfKttLearuWrP9MDH5MBPbIqV92AaeXatLxBI9gBaebbnrfifHhDYfgasaacH8akY=wiFfYdH8Gipec8Eeeu0xXdbba9frFj0=OqFfea0dXdd9vqai=hGuQ8kuc9pgc9s8qqaq=dirpe0xb9q8qiLsFr0=vr0=vr0dc8meaabaqaciaacaGaaeqabaqabeGadaaakeaafaqabeGabaaabaWaaSaaaeaacqWGKbazcqWGwbGvdaWgaaWcbaGaemyAaKgabeaaaOqaaiabdsgaKjabdsha0baacqGH9aqpcqWGgbGrcqGGOaakcqWGwbGvdaWgaaWcbaGaemyAaKgabeaakiabcYcaSiabdkfasnaaBaaaleaacqWGPbqAaeqaaOGaeiykaKIaey4kaSIaemysaK0aaSbaaSqaaiabdMgaPbqabaGccqGHsisliiGacqWF0oazdaWgaaWcbaGaemyAaKgabeaakiabdEgaNnaaBaaaleaacqWGPbqAaeqaaOGaeiikaGIaemOvay1aaSbaaSqaaiabdMgaPbqabaGccqGHsislcqWGfbqrdaWgaaWcbaGaem4CamNaemyEaKNaemOBa4gabeaakiabcMcaPaqaamaalaaabaGaemizaqMaemOuai1aaSbaaSqaaiabdMgaPbqabaaakeaacqWGKbazcqWG0baDaaGaeyypa0Jaem4zaCMaeiikaGIaemOvay1aaSbaaSqaaiabdMgaPbqabaGccqGGSaalcqWGsbGudaWgaaWcbaGaemyAaKgabeaakiabcMcaPaaaaaa@64C4@

where *i *is the index of each neuron, with outer neurons receiving the same current, *I*_1,3 _≡ *I*_*s*_, and the same synaptic strength, *δ*_1 _= *δ*_3 _≡ *δ*. The current input to the middle neuron is higher than to the outer ones: *I*_2 _> *I*_*s*_, leading to the lower uncoupled spiking rate for the outer cells. This was done so that the higher activity of the inner cell drives the outer ones, as might be the case in a typical hierarchical network [3]. The synaptic input to the middle neuron is weaker, *δ*_2 _<*δ*. The synaptic time-constant in Eq. (2) is the same for the outer neurons, *τ*_*syn*1 _= *τ*_*syn*3 _≡ *τ*_*syn*_, but longer for the middle neuron, to model the affect of a slowly-varying modulatory feedback. The conductances, *g*_*i*_, are obtained from Eq. (2), with presynaptic voltage to the middle cell given by: *V*_*pre*2 _= *V*_1 _(*t *- *τ*_*d*_) + *V*_3 _(*t *- *τ*_*d*_), and to the outer cells by *V*_*pre*1,3 _= *V*_2 _(*t *- *τ*_*d*_). The delay in the propagation of the signal is given by *τ*_*d*_.

### 2.1 The effect of delays on correlation and synchronization

**Figure 4:**
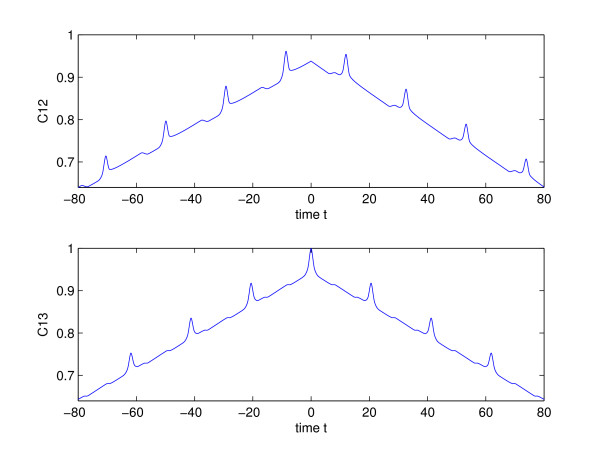
Correlations of spiking output. Top: between the outer and the middle neuron. Bottom: Between outer neurons. *τ*_*d *_= 10 ms, *δ *= 4, *δ*_2 _= 2, *τ*_*syn *_= 1 ms, *τ*_*syn*2 _= 2 ms, *I*_1,3 _= *I*_*s *_= 0.22 nA, *I*_2 _= 0.5 nA.

While the length of the synaptic delay, *τ*_*d*_, does not seem to effect the degree of synchronization, it has a substantial effect on correlations and phase relations between neurons. Figure 4 shows correlations in spiking output between the middle and outer neuron, and between outer neurons for *τ*_*d *_= 10. This delay time is similar, for instance, to the propagation delay between the two hemispheres of the brain [1]. The *x*-axis indicates the time-shift at which the correlations function was computed. The outer neurons are synchronized, since *C*_13 _= 1 at *t *= 0. It can be seen that the greatest correlations between the inner and outer neurons occur when *t *is shifted by the delay time, *τ*_*d*_. The delay also creates spikes in correlation at intervals of twice the delay time, as can be seen in Fig. 4. Since, in this case, the outer neurons are synchronized, the spikes indicate that there are self-correlations in the time series of a single neuron at intervals of 2*τ*_*d*_. This is the round-trip or feed-back time, since its the minimum time that it would take for a signal to travel from one of the neurons, affect the target, and get back to that same neuron. It follows that time delays lead to self-correlations in the spike trains that are not observed when delays are absent, and which may lead to more regular patterns in the time-series data.

For reasons explained in the introduction, *δ *> *δ*_2 _and *τ*_*syn *_<*τ*_*syn*2 _were used to model the affect of a stronger forward and a weaker modulatory feedback. For this type of coupling, the middle neuron leads the outer ones by the delay time, *τ*_*d*_. This type of phase-locking behavior has been observed in other time-delay systems, such as lasers [17,18]. Thus the time-shift between two correlated spike trains should be directly related to the delays in transmission, with the input leading the output by the delay time.

### 2.2 The effect of the coupling strength on firing rate and synchronization

**Figure 5:**
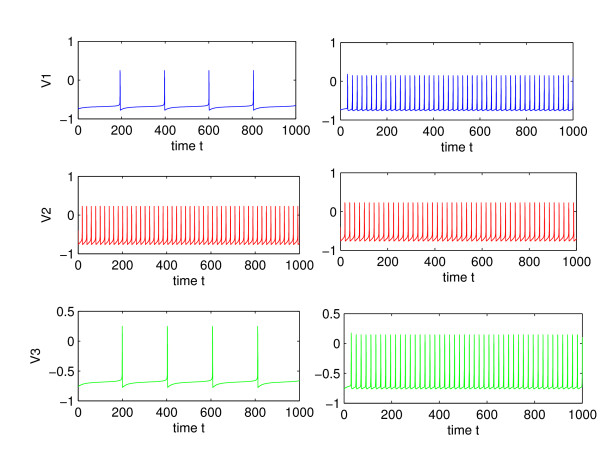
Left: spiking in the absence of coupling. Right: coupling, *δ *= 4, *δ*_2 _= 2, *τ*_*syn *_= 1, *τ*_*syn*2 _= 2. In both cases, *I*_*s *_= 0.22, *I*_2 _= 0.5.

**Figure 6:**
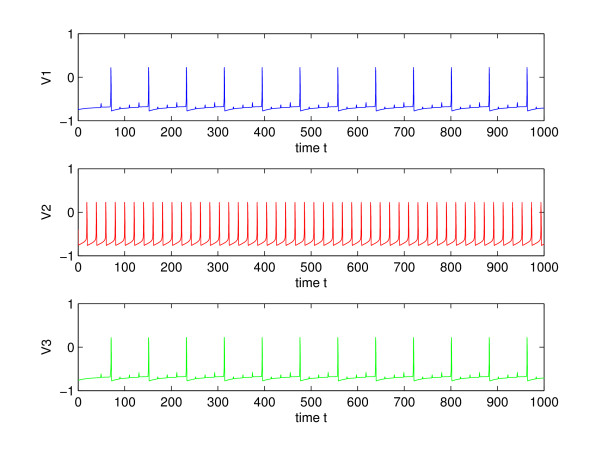
Spiking voltage for weak coupling, *δ *= 0.5, *τ*_*syn *_= 0.03, *δ*_2 _= 0.3. There is 1 : 4 frequency locking between the outer and the middle neurons.

**Figure 7:**
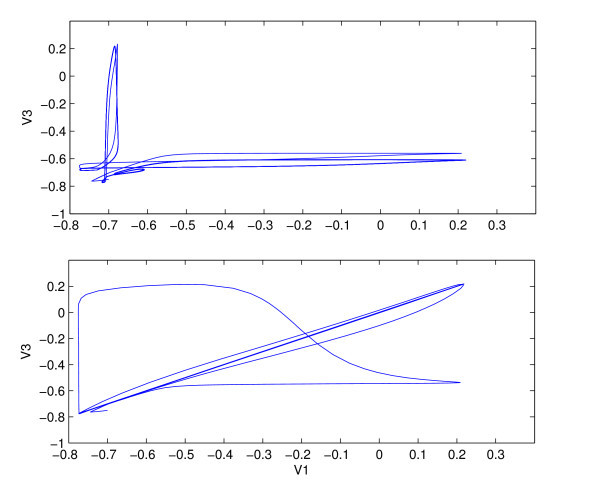
Sensitive dependence of synchronization on coupling strength. Top: *δ *= 1.02, Bottom: *δ *= 1.03, *τ*_*syn *_= 0.5, *δ*_2 _= 0.3.

**Figure 8:**
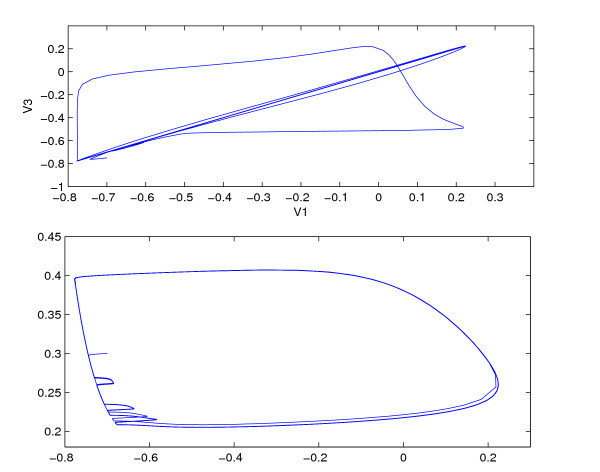
Synchronization for weakly coupled, fast synapses. *δ *= 0.5, *τ*_*syn *_= 0.03, *δ*_2 _= 0.3. Top: synchronization after the transients die out. Bottom: Limit cycle of one of the outer neurons. Same parameters as in Fig. 6.

Increasing the synaptic coupling in general increases the firing rate of the neurons for excitatory synapses. Above a certain value of the coupling strength, there is phase-locking between the inner and the outer neurons, whereby all firing rates are equal. The bifurcation value of synaptic coupling at which 1 : 1 frequency locking occurs depends on the current injected into each cell. In the absence of coupling, a significant difference in injected current for the outer and inner neurons, (*I*_*s *_= 0.22 and *I*_2 _= 0.5 in simulations) leads to a big difference in firing rate. Figure 5 shows a typical voltage trace for the uncoupled, *δ *= 0, and coupled neurons, *δ *= 4, where coupling is sufficiently strong to cause phase-locking. Phase-locking or 1 : 1 frequency locking occurs for *δ *> 3.4. This type of behavior has been observed for mutually coupled neurons in the absence of delays [14]. For lesser values of the coupling strength, different frequency locked behaviors are observed, with frequency ratio between 1 and *f*_*i *_/*f*_*o*_, where *f*_*i *_and *f*_*o *_are the frequencies of inner and outer neurons, respectively, in the absence of synaptic coupling. Figure 6 shows 1 : 4 frequency locking that occurs for weak synaptic coupling: *δ *= 0.5, *δ*_2 _= 0.3. When the coupling is too weak, the outer neurons become desynchronized, as shown in Figure 7. The bifurcation value of *δ*, however depends on the synaptic time constant, *τ*_*syn*_, of the outer-neurons. Thus Figs. 6 and 8, which have a very short time constant of *τ*_*syn *_= 0.03, show synchronization at a lower coupling strength of *δ *= 0.5, compared to a minimum *δ *= 1.03 needed for synchronization of neurons in Fig. 7, where *τ*_*syn *_= 0.5 (a more realistic value for fast synapses). This sensitive dependence of synchronization on the coupling strength and synaptic time constant of the outer-neurons is explored analytically in Section III.

### 2.3 The effect of synaptic time-constant on synchronization and firing rate

**Figure 9:**
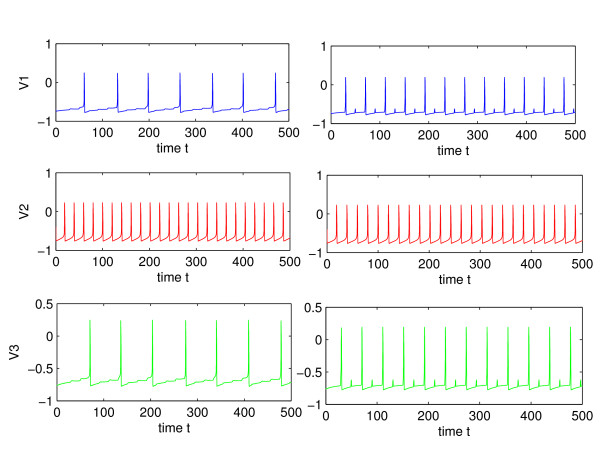
Increase in firing rate as *τ*_*syn *_decreases. Left: *τ*_*syn *_= 2. Right: *τ*_*syn *_= 0.1. In both cases, *δ *= 1, *δ*_2 _= 0.2, *τ*_*syn*2 _= 4, *I*_*s *_= 0.22, *I*_2 _= 0.5.

**Figure 10:**
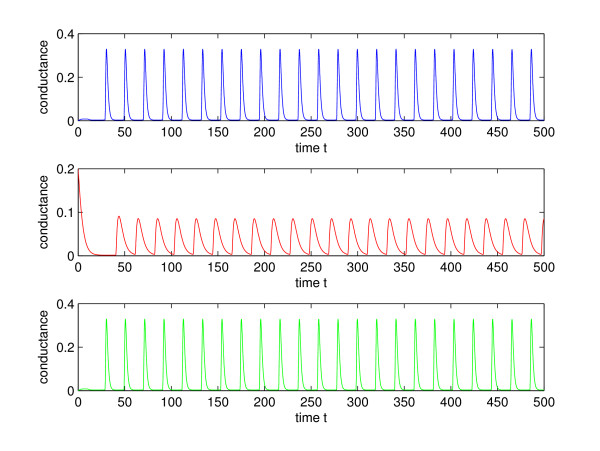
Conductance for *τ*_*syn *_= 1, *τ*_*syn*2 _= 3. All else as in Fig. 4.

Figure 9 shows an increase in firing rate of the outer neurons as their synaptic time constant, *τ*_*syn*_, is decreased. This increase in firing rate may be surprising, since the time constant only controls the width of the conductance spike and not the area under the curve, as shown in Fig. 3. Thus the contribution of a pre-synaptic spike to a change in post-synaptic voltage during an inter-spike interval is largely independent of the synaptic time constant. This can be seen by integrating the *g *(*V*_*i *_- *E*_*syn*_) term in Eq. (4) over the interval of conductance change. Figure 10 shows a fluctuation in conductance for a system given by Eqs. (2) and (4), with *τ*_*syn *_= 1 and *τ*_*syn*2 _= 3. A lower firing rate that occurs for slower synapses may be the result of the decay of any increase in voltage during the inter-spike interval back to the limit cycle trajectory (see the bottom of Fig. 8). A pre-synaptic spike from the inner neuron can trigger a spike from the outer one, when it is delivered toward the end of the inter-spike interval. Thus a more narrow jump in conductance and the resultant jump in voltage, *V*_1,3_, may mean that there is less decay before the critical threshold is reached, thereby increasing the likelihood of a spike.

**Figure 11:**
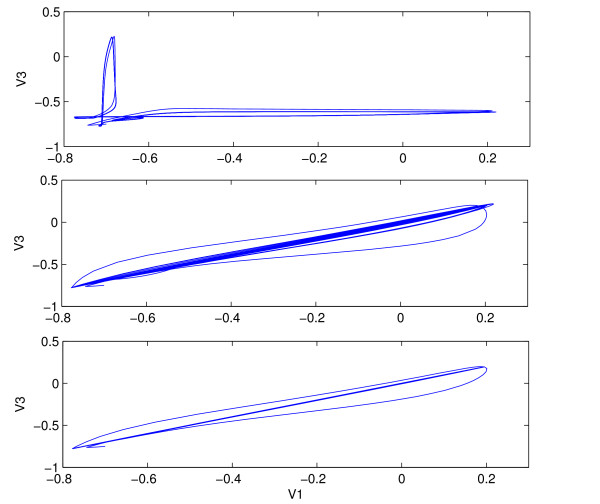
Dependence of synchronization on the synaptic time constant, *τ*_*syn*_. Top: *τ*_*syn *_= 0.5, Middle: *τ*_*syn *_= 0.3, Bottom: *τ*_*syn *_= 0.2, In all three cases, *δ *= 1, *δ*_2 _= 0.

The most noticeable affect of the decrease in *τ *is greater synchronization. This is shown in Fig. 11, where the outer neurons become progressively synchronized as *τ*_*syn *_is decreased from 0.5 to 0.2. It can be seen that for relatively weak coupling of *δ *= 1, *δ*_2 _= 0, and *τ*_*syn *_= 0.2, the outer neurons are completely synchronized, after the transients die out. The next section analyzes the affect of a presynaptic spike on a fast synapse (small *τ*_*syn*_) in synchronizing two nearby trajectories of the outer neurons.

## 3 Analysis of synchronization for weakly coupled, fast synapses

The three neuron model described by Eqs. (2) and (4) possesses internal symmetry, e.g. its equations of motion do not change if the variables {*V*_1_, *R*_1_} and {*V*_3_, *R*_3_} are interchanged. It follows that the synchronized regime, where the symmetric variables are exactly equal: {*V*_1 _= *V*_3 _= *V*_0_, *R*_1 _= *R*_3 _= *R*_0_}, is a solution [19]. For an uncoupled system, this solution would not be a stable one, since any perturbation along the limit cycle would result in a phase-difference. A spiking input, *V*_2_, from the middle neuron stabilized the synchronous state when the synaptic time-constant, *τ*_*syn *_is sufficiently short. To study the affect of a single pre-synaptic spike on two identical neurons, with nearby trajectories along the limit cycle, introduce new variables: V˜
 MathType@MTEF@5@5@+=feaafiart1ev1aaatCvAUfKttLearuWrP9MDH5MBPbIqV92AaeXatLxBI9gBaebbnrfifHhDYfgasaacH8akY=wiFfYdH8Gipec8Eeeu0xXdbba9frFj0=OqFfea0dXdd9vqai=hGuQ8kuc9pgc9s8qqaq=dirpe0xb9q8qiLsFr0=vr0=vr0dc8meaabaqaciaacaGaaeqabaqabeGadaaakeaacuWGwbGvgaacaaaa@2DF0@ = *V*_1 _- *V*_3 _and R˜
 MathType@MTEF@5@5@+=feaafiart1ev1aaatCvAUfKttLearuWrP9MDH5MBPbIqV92AaeXatLxBI9gBaebbnrfifHhDYfgasaacH8akY=wiFfYdH8Gipec8Eeeu0xXdbba9frFj0=OqFfea0dXdd9vqai=hGuQ8kuc9pgc9s8qqaq=dirpe0xb9q8qiLsFr0=vr0=vr0dc8meaabaqaciaacaGaaeqabaqabeGadaaakeaacuWGsbGugaacaaaa@2DE8@ = *R*_1 _- *R*_3_. These new variables correspond to a perturbation transverse to the synchronized state: {*V*_0_, *R*_0_}. Using Eqs. (1), (3) and (4), the linearized dynamics are:

dV˜dt=−n(V0)R˜−h(V0,R0)V˜−δ(ts/τsyn2)exp(−ts/τsyn)V˜
 MathType@MTEF@5@5@+=feaafiart1ev1aaatCvAUfKttLearuWrP9MDH5MBPbIqV92AaeXatLxBI9gBaebbnrfifHhDYfgasaacH8akY=wiFfYdH8Gipec8Eeeu0xXdbba9frFj0=OqFfea0dXdd9vqai=hGuQ8kuc9pgc9s8qqaq=dirpe0xb9q8qiLsFr0=vr0=vr0dc8meaabaqaciaacaGaaeqabaqabeGadaaakeaadaWcaaqaaiabdsgaKjqbdAfawzaaiaaabaGaemizaqMaemiDaqhaaiabg2da9iabgkHiTiabd6gaUjabcIcaOiabdAfawnaaBaaaleaacqaIWaamaeqaaOGaeiykaKIafmOuaiLbaGaacqGHsislcqWGObaAcqGGOaakcqWGwbGvdaWgaaWcbaGaeGimaadabeaakiabcYcaSiabdkfasnaaBaaaleaacqaIWaamaeqaaOGaeiykaKIafmOvayLbaGaacqGHsisliiGacqWF0oazdaqadaqaaiabdsha0naaBaaaleaacqWGZbWCaeqaaOGaei4la8Iae8hXdq3aa0baaSqaaiabdohaZjabdMha5jabd6gaUbqaaiabikdaYaaaaOGaayjkaiaawMcaaGqaciab+vgaLjab+Hha4jab+bhaWnaabmaabaGaeyOeI0IaemiDaq3aaSbaaSqaaiabdohaZbqabaGccqGGVaWlcqWFepaDdaWgaaWcbaGaem4CamNaemyEaKNaemOBa4gabeaaaOGaayjkaiaawMcaaiqbdAfawzaaiaaaaa@66FA@

dR˜dt=15.6(−R˜−m(V0)V˜)
 MathType@MTEF@5@5@+=feaafiart1ev1aaatCvAUfKttLearuWrP9MDH5MBPbIqV92AaeXatLxBI9gBaebbnrfifHhDYfgasaacH8akY=wiFfYdH8Gipec8Eeeu0xXdbba9frFj0=OqFfea0dXdd9vqai=hGuQ8kuc9pgc9s8qqaq=dirpe0xb9q8qiLsFr0=vr0=vr0dc8meaabaqaciaacaGaaeqabaqabeGadaaakeaadaWcaaqaaiabdsgaKjqbdkfaszaaiaaabaGaemizaqMaemiDaqhaaiabg2da9maalaaabaGaeGymaedabaGaeGynauJaeiOla4IaeGOnaydaamaabmaabaGaeyOeI0IafmOuaiLbaGaacqGHsislcqWGTbqBcqGGOaakcqWGwbGvdaWgaaWcbaGaeGimaadabeaakiabcMcaPiqbdAfawzaaiaaacaGLOaGaayzkaaaaaa@4238@

where *n*(*V*_0_) = 26 (*V*_0 _+ 0.95), *h *(*V*_0_, *R*_0_) = (-5.03 + 101.4V02
 MathType@MTEF@5@5@+=feaafiart1ev1aaatCvAUfKttLearuWrP9MDH5MBPbIqV92AaeXatLxBI9gBaebbnrfifHhDYfgasaacH8akY=wiFfYdH8Gipec8Eeeu0xXdbba9frFj0=OqFfea0dXdd9vqai=hGuQ8kuc9pgc9s8qqaq=dirpe0xb9q8qiLsFr0=vr0=vr0dc8meaabaqaciaacaGaaeqabaqabeGadaaakeaacqWGwbGvdaqhaaWcbaGaeGimaadabaGaeGOmaidaaaaa@2FEE@ - 32.45*V*_0 _+ 26*R*_0_), and *m*(*V*_0_) = -(3.8 + 6.6*V*_0_). *t*_*s *_denotes the time since the arrival of the last spike from the center to outer neurons. Equation (5) is valid for a short synaptic time constant, when only the effect of the last presynaptic spike is significant (see Fig. 3). During an inter-spike interval of the outer cells, *n*, *h *and *m *are all positive. This can be easily confirmed by using Fig. 2, where -0.8 <*V *< -0.6 and .18 <*R *< 0.4 during the inter-spike interval, and calculating the lowest possible values of *n*, *h *and *m *for a given range of *V*_0 _and *R*_0_. Since {V˜
 MathType@MTEF@5@5@+=feaafiart1ev1aaatCvAUfKttLearuWrP9MDH5MBPbIqV92AaeXatLxBI9gBaebbnrfifHhDYfgasaacH8akY=wiFfYdH8Gipec8Eeeu0xXdbba9frFj0=OqFfea0dXdd9vqai=hGuQ8kuc9pgc9s8qqaq=dirpe0xb9q8qiLsFr0=vr0=vr0dc8meaabaqaciaacaGaaeqabaqabeGadaaakeaacuWGwbGvgaacaaaa@2DF0@, R˜
 MathType@MTEF@5@5@+=feaafiart1ev1aaatCvAUfKttLearuWrP9MDH5MBPbIqV92AaeXatLxBI9gBaebbnrfifHhDYfgasaacH8akY=wiFfYdH8Gipec8Eeeu0xXdbba9frFj0=OqFfea0dXdd9vqai=hGuQ8kuc9pgc9s8qqaq=dirpe0xb9q8qiLsFr0=vr0=vr0dc8meaabaqaciaacaGaaeqabaqabeGadaaakeaacuWGsbGugaacaaaa@2DE8@} denote the difference between the two nearby trajectories along the limit cycle, there is a relationship between the two variables given by

R˜(t)=−l(V0)V˜(t)
 MathType@MTEF@5@5@+=feaafiart1ev1aaatCvAUfKttLearuWrP9MDH5MBPbIqV92AaeXatLxBI9gBaebbnrfifHhDYfgasaacH8akY=wiFfYdH8Gipec8Eeeu0xXdbba9frFj0=OqFfea0dXdd9vqai=hGuQ8kuc9pgc9s8qqaq=dirpe0xb9q8qiLsFr0=vr0=vr0dc8meaabaqaciaacaGaaeqabaqabeGadaaakeaacuWGsbGugaacaiabcIcaOiabdsha0jabcMcaPiabg2da9iabgkHiTiabdYgaSjabcIcaOiabdAfawnaaBaaaleaacqaIWaamaeqaaOGaeiykaKIafmOvayLbaGaacqGGOaakcqWG0baDcqGGPaqkaaa@3CD1@

where *l *is the negative of the slope of the limit cycle at {*V*_0_(*t*), *R*_0_(*t*)}. From Fig. 2, *l*(*V*_0_) ≈ 2 during the inter-spike interval. The above equation is valid as long as two nearby trajectories remain on the limit cycle.

The dynamics of V˜
 MathType@MTEF@5@5@+=feaafiart1ev1aaatCvAUfKttLearuWrP9MDH5MBPbIqV92AaeXatLxBI9gBaebbnrfifHhDYfgasaacH8akY=wiFfYdH8Gipec8Eeeu0xXdbba9frFj0=OqFfea0dXdd9vqai=hGuQ8kuc9pgc9s8qqaq=dirpe0xb9q8qiLsFr0=vr0=vr0dc8meaabaqaciaacaGaaeqabaqabeGadaaakeaacuWGwbGvgaacaaaa@2DF0@ along a limit cycle can now be calculated by substituting Eq. (7) into Eq. (5), dividing by V˜
 MathType@MTEF@5@5@+=feaafiart1ev1aaatCvAUfKttLearuWrP9MDH5MBPbIqV92AaeXatLxBI9gBaebbnrfifHhDYfgasaacH8akY=wiFfYdH8Gipec8Eeeu0xXdbba9frFj0=OqFfea0dXdd9vqai=hGuQ8kuc9pgc9s8qqaq=dirpe0xb9q8qiLsFr0=vr0=vr0dc8meaabaqaciaacaGaaeqabaqabeGadaaakeaacuWGwbGvgaacaaaa@2DF0@, bringing *dt *over to the right-hand side of the equation and integrating. For a small synaptic time constant, *τ*_*syn *_≤ 1, the width of a duration of a post-synaptic spike, Δ*t*, is short compared to the time-scale of neuronal dynamics of outer cells during the inter-spike interval. Here, Δ*t *measures the duration of a conductance spike, (*t*_*s*_/τsyn2
 MathType@MTEF@5@5@+=feaafiart1ev1aaatCvAUfKttLearuWrP9MDH5MBPbIqV92AaeXatLxBI9gBaebbnrfifHhDYfgasaacH8akY=wiFfYdH8Gipec8Eeeu0xXdbba9frFj0=OqFfea0dXdd9vqai=hGuQ8kuc9pgc9s8qqaq=dirpe0xb9q8qiLsFr0=vr0=vr0dc8meaabaqaciaacaGaaeqabaqabeGadaaakeaaiiGacqWFepaDdaqhaaWcbaGaem4CamNaemyEaKNaemOBa4gabaGaeGOmaidaaaaa@33E6@) *exp *(-*t*_*s*_/*τ*_*syn*_), which is quite narrow for sufficiently fast synapses (see Fig. 3). Thus most of the change in V˜
 MathType@MTEF@5@5@+=feaafiart1ev1aaatCvAUfKttLearuWrP9MDH5MBPbIqV92AaeXatLxBI9gBaebbnrfifHhDYfgasaacH8akY=wiFfYdH8Gipec8Eeeu0xXdbba9frFj0=OqFfea0dXdd9vqai=hGuQ8kuc9pgc9s8qqaq=dirpe0xb9q8qiLsFr0=vr0=vr0dc8meaabaqaciaacaGaaeqabaqabeGadaaakeaacuWGwbGvgaacaaaa@2DF0@ during the narrow post-synaptic spike is due to the spike itself. This can be seen by considering a voltage difference of two nearby trajectories at time *t *+ Δ*t *in the **absence **of synaptic input: V˜u(t+Δt)≈V˜(t)exp⁡(Δt(nl−h))
 MathType@MTEF@5@5@+=feaafiart1ev1aaatCvAUfKttLearuWrP9MDH5MBPbIqV92AaeXatLxBI9gBaebbnrfifHhDYfgasaacH8akY=wiFfYdH8Gipec8Eeeu0xXdbba9frFj0=OqFfea0dXdd9vqai=hGuQ8kuc9pgc9s8qqaq=dirpe0xb9q8qiLsFr0=vr0=vr0dc8meaabaqaciaacaGaaeqabaqabeGadaaakeaacuWGwbGvgaacamaaBaaaleaacqWG1bqDaeqaaOGaeiikaGIaemiDaqNaey4kaSIaeuiLdqKaemiDaqNaeiykaKIaeyisISRafmOvayLbaGaacqGGOaakcqWG0baDcqGGPaqkcyGGLbqzcqGG4baEcqGGWbaCcqGGOaakcqqHuoarcqWG0baDcqGGOaakcqWGUbGBcqWGSbaBcqGHsislcqWGObaAcqGGPaqkcqGGPaqkaaa@4C08@, with *n *and *h *defined after Eq. (6) and *l *given in Eq. (7). This expression was obtained by substituting Eq. (7) into Eq. (5) and integrating over Δ*t *while assuming that *V*_0 _stays almost constant over Δ*t*. Since Δ*t *is small, we have |Δ*t *(*nl *- *h*)| ≪ {1, *δ*}. So that only the conductance term in Eq. (5) makes a significant contribution to V˜
 MathType@MTEF@5@5@+=feaafiart1ev1aaatCvAUfKttLearuWrP9MDH5MBPbIqV92AaeXatLxBI9gBaebbnrfifHhDYfgasaacH8akY=wiFfYdH8Gipec8Eeeu0xXdbba9frFj0=OqFfea0dXdd9vqai=hGuQ8kuc9pgc9s8qqaq=dirpe0xb9q8qiLsFr0=vr0=vr0dc8meaabaqaciaacaGaaeqabaqabeGadaaakeaacuWGwbGvgaacaaaa@2DF0@ during the duration, Δ*t*, of a post-synaptic spike. Dividing Eq. (5) by V˜
 MathType@MTEF@5@5@+=feaafiart1ev1aaatCvAUfKttLearuWrP9MDH5MBPbIqV92AaeXatLxBI9gBaebbnrfifHhDYfgasaacH8akY=wiFfYdH8Gipec8Eeeu0xXdbba9frFj0=OqFfea0dXdd9vqai=hGuQ8kuc9pgc9s8qqaq=dirpe0xb9q8qiLsFr0=vr0=vr0dc8meaabaqaciaacaGaaeqabaqabeGadaaakeaacuWGwbGvgaacaaaa@2DF0@ and integrating, we get

V˜(t+Δt)≈V˜(t)e−δ
 MathType@MTEF@5@5@+=feaafiart1ev1aaatCvAUfKttLearuWrP9MDH5MBPbIqV92AaeXatLxBI9gBaebbnrfifHhDYfgasaacH8akY=wiFfYdH8Gipec8Eeeu0xXdbba9frFj0=OqFfea0dXdd9vqai=hGuQ8kuc9pgc9s8qqaq=dirpe0xb9q8qiLsFr0=vr0=vr0dc8meaabaqaciaacaGaaeqabaqabeGadaaakeaacuWGwbGvgaacaiabcIcaOiabdsha0jabgUcaRiabfs5aejabdsha0jabcMcaPiabgIKi7kqbdAfawzaaiaGaeiikaGIaemiDaqNaeiykaKIaemyzau2aaWbaaSqabeaacqGHsisliiGacqWF0oazaaaaaa@3EFD@

The above equation gives a change in V˜
 MathType@MTEF@5@5@+=feaafiart1ev1aaatCvAUfKttLearuWrP9MDH5MBPbIqV92AaeXatLxBI9gBaebbnrfifHhDYfgasaacH8akY=wiFfYdH8Gipec8Eeeu0xXdbba9frFj0=OqFfea0dXdd9vqai=hGuQ8kuc9pgc9s8qqaq=dirpe0xb9q8qiLsFr0=vr0=vr0dc8meaabaqaciaacaGaaeqabaqabeGadaaakeaacuWGwbGvgaacaaaa@2DF0@ following a synaptic change in conductance. Using Eqs. (6)-(8), the dynamics of R˜
 MathType@MTEF@5@5@+=feaafiart1ev1aaatCvAUfKttLearuWrP9MDH5MBPbIqV92AaeXatLxBI9gBaebbnrfifHhDYfgasaacH8akY=wiFfYdH8Gipec8Eeeu0xXdbba9frFj0=OqFfea0dXdd9vqai=hGuQ8kuc9pgc9s8qqaq=dirpe0xb9q8qiLsFr0=vr0=vr0dc8meaabaqaciaacaGaaeqabaqabeGadaaakeaacuWGsbGugaacaaaa@2DE8@ immediately following a narrow post-synaptic spike can be approximated as

dR˜dt=15.6(−1+(m(V0)l(V0))e−δ)R˜
 MathType@MTEF@5@5@+=feaafiart1ev1aaatCvAUfKttLearuWrP9MDH5MBPbIqV92AaeXatLxBI9gBaebbnrfifHhDYfgasaacH8akY=wiFfYdH8Gipec8Eeeu0xXdbba9frFj0=OqFfea0dXdd9vqai=hGuQ8kuc9pgc9s8qqaq=dirpe0xb9q8qiLsFr0=vr0=vr0dc8meaabaqaciaacaGaaeqabaqabeGadaaakeaadaWcaaqaaiabdsgaKjqbdkfaszaaiaaabaGaemizaqMaemiDaqhaaiabg2da9maalaaabaGaeGymaedabaGaeGynauJaeiOla4IaeGOnaydaamaabmaabaGaeyOeI0IaeGymaeJaey4kaSYaaeWaaeaadaWcaaqaaiabd2gaTjabcIcaOiabdAfawnaaBaaaleaacqaIWaamaeqaaOGaeiykaKcabaGaemiBaWMaeiikaGIaemOvay1aaSbaaSqaaiabicdaWaqabaGccqGGPaqkaaaacaGLOaGaayzkaaGaemyzau2aaWbaaSqabeaacqGHsisliiGacqWF0oazaaaakiaawIcacaGLPaaacuWGsbGugaacaaaa@4D01@

From Eq. (8), a single presynaptic spike from a center neuron acts to decrease the voltage difference, V˜
 MathType@MTEF@5@5@+=feaafiart1ev1aaatCvAUfKttLearuWrP9MDH5MBPbIqV92AaeXatLxBI9gBaebbnrfifHhDYfgasaacH8akY=wiFfYdH8Gipec8Eeeu0xXdbba9frFj0=OqFfea0dXdd9vqai=hGuQ8kuc9pgc9s8qqaq=dirpe0xb9q8qiLsFr0=vr0=vr0dc8meaabaqaciaacaGaaeqabaqabeGadaaakeaacuWGwbGvgaacaaaa@2DF0@, of the outer neurons by a factor of *exp*(-*δ*). This presynaptic spike also decreases R˜
 MathType@MTEF@5@5@+=feaafiart1ev1aaatCvAUfKttLearuWrP9MDH5MBPbIqV92AaeXatLxBI9gBaebbnrfifHhDYfgasaacH8akY=wiFfYdH8Gipec8Eeeu0xXdbba9frFj0=OqFfea0dXdd9vqai=hGuQ8kuc9pgc9s8qqaq=dirpe0xb9q8qiLsFr0=vr0=vr0dc8meaabaqaciaacaGaaeqabaqabeGadaaakeaacuWGsbGugaacaaaa@2DE8@ by decreasing the positive contribution from the *m*/*l *> 0 term in Eq. (9). Thus the immediate effect of a presynaptic input is to decrease the perturbation from synchronized state of the outer neurons, pushing their trajectories closer in phase-space.

Eqs. (8) and (9) show that a spiking input from the middle neuron has a stabilizing affect on the synchronized state when the synaptic time constant is short. Since the difference in trajectories, V˜
 MathType@MTEF@5@5@+=feaafiart1ev1aaatCvAUfKttLearuWrP9MDH5MBPbIqV92AaeXatLxBI9gBaebbnrfifHhDYfgasaacH8akY=wiFfYdH8Gipec8Eeeu0xXdbba9frFj0=OqFfea0dXdd9vqai=hGuQ8kuc9pgc9s8qqaq=dirpe0xb9q8qiLsFr0=vr0=vr0dc8meaabaqaciaacaGaaeqabaqabeGadaaakeaacuWGwbGvgaacaaaa@2DF0@, R˜
 MathType@MTEF@5@5@+=feaafiart1ev1aaatCvAUfKttLearuWrP9MDH5MBPbIqV92AaeXatLxBI9gBaebbnrfifHhDYfgasaacH8akY=wiFfYdH8Gipec8Eeeu0xXdbba9frFj0=OqFfea0dXdd9vqai=hGuQ8kuc9pgc9s8qqaq=dirpe0xb9q8qiLsFr0=vr0=vr0dc8meaabaqaciaacaGaaeqabaqabeGadaaakeaacuWGsbGugaacaaaa@2DE8@ is taken along a limit cycle, the maximum Lyapunov exponent in the absence of synaptic coupling would be zero (corresponding to the displacement along a trajectory in phase-space), and the transverse exponents must be negative since the trajectory collapses onto a limit cycle. It follows that, for sufficiently small *τ*_*syn*_, a common synaptic input acting during the inter-spike interval should eventually synchronize the neurons, even for weak synaptic coupling.

Figure 8 shows the synchronization of the outer neurons for a very short synaptic time constant, *τ*_*syn *_= 0.03 and weak coupling, *δ *= 0.5. After the transients die out, the outer neurons become synchronized, thereby falling on a straight line in the *V*_1 _vs *V*_3 _plot. As can be seen in Figure 6, for weak coupling, there is a big difference in firing rate between the outer and the middle neuron, due to differences in injected current. It follows that the synchronization is not due to phase-locking between the middle and the outer cells. The effect of the spiking input on the limit cycle trajectory can be seen at the bottom of Figure 8. There is an integer ratio between the inner and the outer frequencies, whereby the onset of a spike in the outer neuron is triggered by spiking input from the middle one, delivered toward the end of the inter-spike interval. The phenomena is similar to the subharmonic resonance where the limit cycle responds at a subharmonic of the stimulus frequency [14].

Eqs. (8) and (9) show a sensitive dependence of synchronization on the coupling strength from the middle to the outer neurons. This is confirmed by numerics. Figure 7 shows *V*_1 _vs *V*_3 _for two slightly different values of *δ*, *δ *= 1.02 and *δ *= 1.03. A slight change in *δ *leads to an onset of synchronization between the outer neurons.

## 4 Conclusion

Synchronization of nearby cells is often the result of receiving common input, such as when a pyramidal cell sends projections to a targeted area in the cortex [3]. While pyramidal cells tend to target specific areas, matrix projection cells from the thalamus reach in a diffuse manner into adjacent cortical areas helping to synchronize the activity of large populations of cells [3,20]. The three neuron scheme investigated in this paper is a simple model for studying these types of hierarchical networks, since it incorporates this phenomena of synchronization of certain areas of the brain (outer neurons) due to common input from a different area (middle neuron), and since synchronization of the two outer neurons would indicate synchronization of many neurons, if coupled to the middle neuron in the same way as the two outer neurons in a three neuron model.

This model of three mutually coupled cortical neurons with delays was studied using analysis and numerical simulation. The outer neurons were stimulated with smaller current and had a much lower firing frequency in the uncoupled case, with their frequency significantly increasing depending on the strength of synaptic coupling with the middle neuron. At higher values of the synaptic coupling constant, typical phase-locked behavior and 1 : 1 frequency locking was found between the middle and the outer neurons, with different frequency locking ratios as the synaptic strength was lowered. It was found that delays affected the time-series data by introducing correlations at the time-scale of the delay. While the spiking behavior in the synchronized case was fairly regular, this effect would be interesting to explore for a more complicated, chaotic spike train that can be achieved by incorporating slow adaptation currents into the neuron model. In the case of phase or frequency locking, the middle neuron leads the outer by the delay time, *τ*_*d*_.

While synchronization of outer neurons was sensitive to the synaptic strength, the synaptic time constant of outer neurons, *τ*_*syn *_was also highly significant. It was found that shorter synaptic constant substantially improves correlations, leading to zero-lag synchronization of end neurons even when the coupling strength is very weak. A short synaptic constant was also able to significantly increase firing rate, to the point of inducing 1 : 1 frequency locking with the middle neuron, at a much weaker mutual coupling than would otherwise occur for the input currents used. Analysis of dynamics for fast synapses showed that fast synaptic input during the inter-spike interval stabilized the synchronization manifold, even for arbitrarily weak coupling, and independent of the phase relationship between the inner and outer cells. This indicates that even a very weak synaptic input can synchronize cells, as long as the synaptic time constant is suficiently short. The finding may have significance in synchronizing large groups of cells in the cortex via weak synaptic input from other areas, such as the thalamus, or other areas in the cortex proper.
